# Cellular Stresses and Stress Responses in the Pathogenesis of Insulin Resistance

**DOI:** 10.1155/2018/4321714

**Published:** 2018-07-09

**Authors:** Arnold N. Onyango

**Affiliations:** Department of Food Science and Technology, Jomo Kenyatta University of Agriculture and Technology, P.O. Box 62000, Nairobi 00200, Kenya

## Abstract

Insulin resistance (IR), a key component of the metabolic syndrome, precedes the development of diabetes, cardiovascular disease, and Alzheimer's disease. Its etiological pathways are not well defined, although many contributory mechanisms have been established. This article summarizes such mechanisms into the hypothesis that factors like nutrient overload, physical inactivity, hypoxia, psychological stress, and environmental pollutants induce a network of cellular stresses, stress responses, and stress response dysregulations that jointly inhibit insulin signaling in insulin target cells including endothelial cells, hepatocytes, myocytes, hypothalamic neurons, and adipocytes. The insulin resistance-inducing cellular stresses include oxidative, nitrosative, carbonyl/electrophilic, genotoxic, and endoplasmic reticulum stresses; the stress responses include the ubiquitin-proteasome pathway, the DNA damage response, the unfolded protein response, apoptosis, inflammasome activation, and pyroptosis, while the dysregulated responses include the heat shock response, autophagy, and nuclear factor erythroid-2-related factor 2 signaling. Insulin target cells also produce metabolites that exacerbate cellular stress generation both locally and systemically, partly through recruitment and activation of myeloid cells which sustain a state of chronic inflammation. Thus, insulin resistance may be prevented or attenuated by multiple approaches targeting the different cellular stresses and stress responses.

## 1. Introduction

The hormone insulin plays an important role in maintaining physiological levels of blood glucose, through various effects on insulin target cells. In endothelial cells, it promotes the release of nitric oxide and endothelin, which, respectively, promote vasodilation and vasoconstriction, and the combined vasodilatory and vasoconstrictive effects improve the distribution of blood glucose to target organs such as skeletal muscles [1]. It promotes glycogen synthesis in hepatocytes, skeletal myocytes, and adipocytes [2, 3], downregulates the expression of gluconeogenetic enzymes in hepatocytes, and promotes glucose uptake through the GLUT 4 receptor in skeletal myocytes and adipocytes [2, 3]. In specific types of hypothalamic neurons, it inhibits the expression of orexigenic neuropeptides such as neuropeptide Y (NYP) or agouti-related peptide (AgRP) and thereby contributes to decreased food intake [4–8]. Insulin also inhibits food intake by promoting expression of anorexigenic neuropeptides such as proopiomelanocorticotropin (POMC) and cocaine- and amphetamine-regulated transcript (CaRT) in the arcuate nucleus, which together promote the activity of *α*-melanocyte-stimulating hormone in neurons in the paraventricular nucleus (4–8). Besides inhibiting AgRP synthesis, insulin-induced hyperpolarization of the AgRP-expressing arcuate neurons reduces the firing rate of these neurons and results in the generation and transmission of signals from the motor nucleus of the vagus nerve to the liver, resulting in increased hepatic interleukin 6 (IL-6) production, IL-6-mediated activation of signal transducer and activator of transcription 3 (STAT-3), and STAT-3-mediated decrease in the expression of gluconeogenic genes such as glucose-6-phosphatase and phosphoenol pyruvate carboxykinase (PEPCK) [9–12].

**Figure 1:**
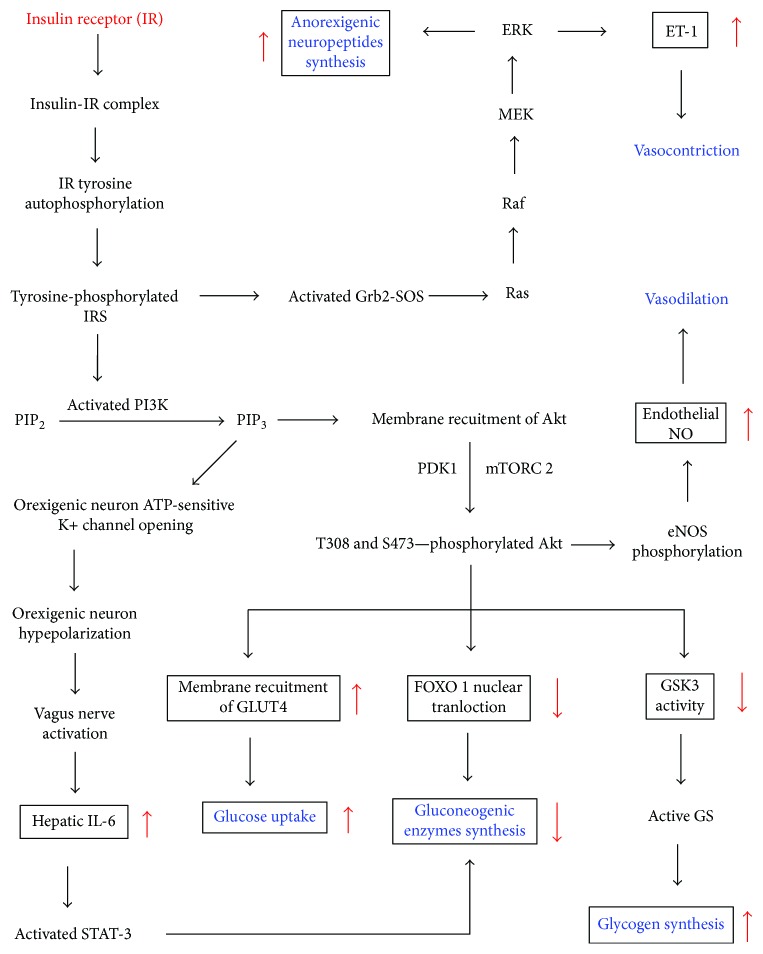
Insulin signaling pathways via the insulin receptor (IR) and insulin receptor substrates (IRS) [1–4, 9–12]. ERK: extracellular signal-regulated kinase; eNOS: endothelial nitric oxide synthase; FOXO: forkhead box O transcription factor; GLUT 4: glucose transporter 4; Grb2-SOS: growth factor receptor-bound 2- (Grb2-) son of sevenless (Sos) protein complex; GS: glycogen synthase; GSK3: glycogen synthase kinase 3; IL-6: interleukin 6; MEK: MAPK (mitogen-activated protein kinase)/ERK kinase; mTORC 2: mammalian target of rapamycin 2; NO: nitric oxide; PDK 1: 3-phosphoinositide-dependent kinase-1; PIP_2_: phosphatidyl inositol 4,5-biphosphate; PIP_3_: phosphatidyl inositol 3,4,5-triphosphate; STAT-3: signal transducer and activator of transcription 3.

Insulin resistance refers to a condition in which insulin-responsive cells undergo a less than normal response to insulin, such as a reduced activation of endothelial nitric oxide synthase in endothelial cells [13]. It involves disruption of specific events in the insulin signaling pathways. Insulin signaling begins with insulin binding to the insulin receptor (IR), a receptor tyrosine kinase, which then undergoes autophosphorylation of various intracellular tyrosine residues, resulting in the recruitment and tyrosine phosphorylation of adaptor proteins including insulin receptor substrates (IRS) such as IRS1 and IRS2 (Figure 1).

Signaling downstream of IRS occurs by several pathways (Figure 1). One such pathway sequentially involves activation of phosphatidyl inositol 3-kinase (PI3K); conversion of phosphatidyl inositol 4,5-biphosphate (PIP2) to phosphatidyl inositol 3,4,5-triphosphate (PIP_3_); recruitment of Akt (protein kinase B (PKB)) to the plasma membrane; phosphorylation of Akt by 3-phosphoinositide-dependent kinase-1 (PDK 1) and mammalian target of rapamycin complex 2 (mTORC 2); and Akt-mediated phosphorylation of a number of downstream protein substrates that induce effects such as activation of glycogen synthase (GS) in adipocytes, skeletal myocytes, and hepatocytes, translocation of glucose transporter 4 (GLUT 4) to the plasma membrane of adipocytes and skeletal myocytes, phosphorylation of the forkhead transcription factor (FOXO 1) to inhibit expression of gluconeogenic enzymes in hepatocytes, or activation of endothelial nitric oxide synthase in endothelial cells [1–3, 13] (Figure 1). Akt also activates mTORC 1 which not only is involved in feedback inhibition of IRS but also inhibits synthesis of orexigenic neuropeptides by hypothalamic neurons (not shown) [14].

In another pathway which involves orexigenic (AgRP-producing) hypothalamic neurons, PI3K promotes opening of ATP-sensitive K+ channels, resulting in sequential hyperpolarization of these neurons, transmission of signals from the vagus nerve to the liver, increased hepatic IL-6 synthesis, activation of STAT-3, and decreased expression of gluconeogenic enzymes (Figure 1) [9–12]. On the other hand, insulin-mediated upregulation of the production of anorexigenic neuropeptides by hypothalamic neurons proceeds through IRS-mediated activation of the growth factor receptor-bound 2- (Grb2-) son of sevenless (Sos) protein complex (Grb2-Sos) and downstream activation of the Ras-Raf-MEK-ERK pathway [4]. In endothelial cells, ERK promotes the synthesis of endothelin-1 [1].

Because insulin resistance contributes to the development of noncommunicable diseases such as diabetes, cardiovascular disease, fatty liver disease, Alzheimer's disease, and impaired lung function [12, 13, 15–18], much effort has been directed toward understanding the mechanisms of its pathogenesis through studies involving cell cultures, animal models, and clinical studies. Cell cultures of hepatocytes, adipocytes, skeletal muscle cells, endothelial cells, or neurons incubated with palmitate or high sugar concentrations develop insulin resistance [19–26]. Some of the cellular events and mechanisms that have been shown to be involved in the development of insulin resistance in these cells both in vitro and in vivo include (i) toll-like receptor 4 (TLR4) and associated inhibitor of kappa B kinase- (IKK-) nuclear factor kappa B (NF-*κ*B) signaling [27–31]; (ii) advanced glycation end products (AGEs) or uric acid-induced receptor for AGE (RAGE) signaling via NF-*κ*B [30, 32–34]; (iii) oxidized low-density lipoprotein- (oxLDL-) mediated RAGE or Lox-1 signaling and the resultant activation of NF-*κ*B and formation of peroxynitrite [30, 35, 36]; (iv) upregulation of NADPH oxidase (Nox) expression and activity [20, 21, 30, 37–39]; (v) increased mitochondrial reactive oxygen species (ROS) generation [30, 40]; (vi) upregulation of inducible nitric oxide synthase (INOS) [30, 41–43]; (vii) increased diacylglycerol synthesis [30, 44]; (viii) increased ceramide synthesis [30, 45, 46]; (ix) activation of protein kinase C (PKC) isoforms [30, 37, 47]; (x) activation of mitogen-activated protein kinases (MAPKs) such as c-Jun N-terminal kinase (JNK), p38 MAPK, and extracellular signal-regulated kinase (ERK) [20, 28, 30]; (xi) endoplasmic reticulum stress and the unfolded protein response [30, 43, 48–50]; (xii) dysregulation of the heat shock response [51–53]; (xiii) autophagy dysregulation [54]; (xiv) apoptosis [55]; (xv) p53 activation [56]; and (xvi) inflammasome activation [57, 58]. Thus, insulin resistance is regarded as a complex disorder that defies a single etiological pathway [59].

**Figure 2:**
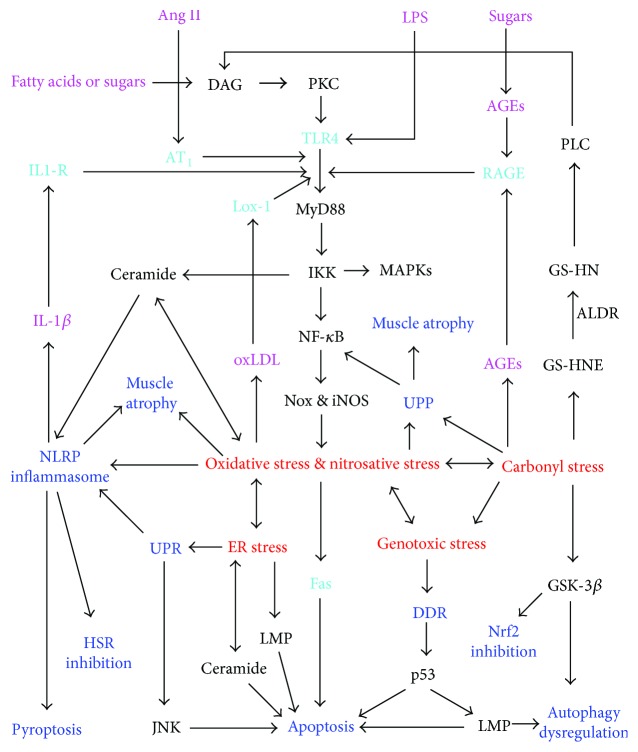
Pathways to interconnected cellular stresses, stress responses, and stress response dysregulations that contribute to insulin resistance in insulin target cells exposed to excess nutrients (sugars or fatty acids such as palmitate), angiotensin II (Ang II), or bacterial lipopolysaccharide (LPS). ALDR: aldose reductase; AGE: advanced glycation end product; AT_1_: angiotensin receptor type 1; DAG: diacylglycerol; DDR: DNA damage response; GS-HNE: glutathione-HNE adduct; GS-HN: glutathionyl-1,4-dihydroxynonene; GSK: glycogen synthase kinase; HSR: heat shock response; IL-1*β*: interleukin 1*β*; IL1-R: interleukin 1 receptor; iNOS: inducible nitric oxide synthase; IKK: inhibitor of kappa B kinase; LMP: lysosomal membrane permeabilization; MAPK: mitogen-activated protein kinase; NF-*κ*B: nuclear factor kappa B; Nox: NADPH oxidase; PKC: protein kinase C; PLC: phospholipase C; TLR4: toll-like receptor 4; UPP: ubiquitin-proteosome pathway; UPR: unfolded protein response. The stresses, stress responses, and signaling pathways generating them contribute to insulin resistance by multiple mechanisms as described in the text. Insulin resistance may occur because of the combined effects of different components of the system, and these components may promote IR to different extents in different cell types.

This review summarizes the above mechanisms into a unifying hypothesis that the pathogenesis of insulin resistance involves generation of oxidative stress, nitrosative stress, carbonyl stress, endoplasmic reticulum stress, and genotoxic stress through interconnected pathways; induction of various responses to these stresses, such as the unfolded protein response (UPR), the ubiquitin proteasome pathway (UPP), DNA damage response (DDR), the NRLP3 inflammasome, and apoptosis; and the dysregulation of stress responses such as autophagy, heat shock response, and nuclear factor erythroid-2-related factor 2 (Nrf2) signaling in insulin target cells (as exemplified in Figure 2). Each of the stresses, stress responses, and stress response dysregulations contributes to insulin resistance in multiple ways.

## 2. Pathways to Cellular Stresses in Insulin Target Cells

### 2.1. Pathways to Oxidative and Nitrosative Stresses in Response to Overnutrition, Physical Inactivity, Hypoxia, Psychological Stress, or Environmental Pollutants

As illustrated in Figure 2, cell surface receptors such as the TLR4, RAGE, Lox-1, and angiotensin receptor type 1 (AT_1_) are involved in signaling pathways that generate oxidative stress and nitrosative stress.

A high-fat or high-fructose diet promotes the growth of gram-negative bacteria in the colon, resulting in endotoxemia and the release of enteric lipopolysaccharide (LPS) into blood plasma [60, 61]. LPS is a direct ligand for TLR4 and induces TLR4-dependent oxidative stress and inhibition of insulin signaling in both peripheral insulin target cells and hypothalamic neurons [28, 30, 62, 63]. TLR4 signaling via MyD88 and IRAK 4 leads to the activation of IKK [28, 30, 64]. One of the most important targets of IKK activation is NF-*κ*B, which, for example, was found to be essential for palmitate-induced insulin resistance in C2C12 skeletal muscle cells [65]. NF-*κ*B induces expression of protein tyrosine phosphatase B (PTPB), a negative regulator of the insulin receptor [66] and proinflammatory genes such as tumor necrosis factor-*α* (TNF-*α*), interleukin 1*β* (IL-1*β*), and interleukin 6 (IL-6) [67]. It also upregulates the expression of Nox and iNOS, which produce superoxide anions (O_2_^−^) and nitric oxide (NO), respectively [28–30, 68, 69]. Superoxide anions are rapidly converted to hydrogen peroxide (H_2_O_2_) by superoxide dismutase (SOD) [70]. Superoxide anions also rapidly react with NO to form peroxynitrite (OONO^−^) [70], which reacts with hydrogen peroxide (H_2_O_2_) to form singlet oxygen (^1^O_2_) [30, 71], which in turn reacts with biomolecules such as lipids and proteins to form organic hydroperoxides (ROOH) [30]. Excessive production of reactive oxygen species (ROS) such as superoxide anions, hydrogen peroxide, organic hydroperoxides, and singlet oxygen results in oxidative stress when the ROS outweigh the cellular antioxidant capacity [72]. Likewise, excessive formation of peroxynitrite results in nitrosative stress. NF-*κ*B-dependent induction of iNOS and Nox may further contribute to mitochondrial oxidative and nitrosative stresses. This is because, even when H_2_O_2_ and NO are generated extramitochondrially, they readily enter the mitochondria and induce electron leakage from the electron transport chain (ETC), thus promoting the generation of mitochondrial superoxide anions, H_2_O_2_, peroxynitrite, singlet oxygen, and lipid hydroperoxides [30, 70].

The nonenzymatic reaction of sugars with proteins (Maillard reaction) leads to the formation of hydrogen peroxide, singlet oxygen, and advanced glycation end products (AGEs) such as glyoxal lysine and methylglyoxal lysine [73, 74]. AGEs accumulate in plasma and tissues of animals and humans on diets rich in fructose or preformed AGEs [75]. AGEs signal via the RAGE receptor to induce activation of NF-*κ*B via some components of the TLR4 pathway and thus produce similar effects as LPS, including the induction of oxidative and nitrosative stresses (Figure 2) [30, 32, 76].

Oversupply of fatty acids to insulin target cells occurs because of excessive dietary intake, obesity, or muscle inactivity-associated decrease in beta oxidation of fatty acids [59, 77, 78]. Palmitate and laurate can induce the activation of IKK, NF-*κ*B, and oxidative stress independently of LPS [79–84]. This at least partly involves increased synthesis of diacylglycerol (DAG), a cofactor of protein kinase C (PKC) isoforms which activate Nox isoforms and NF-*κ*B [30, 59, 80, 82–85]. Similarly, exposure of endothelial cells to high glucose levels results in DAG formation and subsequent activation of PKC and Nox [80]. The activation of Nox and NF-*κ*B by PKC isoforms may involve PKC-induced TLR4 signaling as shown in Figure 2 [30, 64, 81]. However, DAG-PKC-induced insulin resistance without TLR4 activation has also been reported [84].

Palmitate is a substrate of serine palmitoyl transferase (SPT) in the first step of the de novo biosynthesis of the sphingolipid ceramide, an inhibitor of insulin signaling by multiple mechanisms including activation of protein phosphatase 2A (PP2A) and PKC-*ζ*, which promote dephosphorylation of Akt or serine phosphorylation of IRS, respectively [45, 81, 86, 87]. Long-term ceramide action also promotes serine phosphorylation of IRS via sequential activation of the double-stranded RNA-activated protein kinase (PKR) and JNK [88]. Palmitate-induced TLR4-IKK signaling promotes ceramide biosynthesis by upregulating the synthesis of SPT and ceramide synthases (Figure 2) [81, 86]. Ceramide is an important contributor to oxidative stress. It induces mitochondrial superoxide anion and H_2_O_2_ generation by blocking the electron transport system at complex III [89]. Mitochondrial superoxide anions generated in this manner induce opening of the mitochondrial permeability transition pore, allowing mitochondrial ROS to move into the cytoplasm [90]. Some mechanisms by which ceramide induces insulin resistance, such as apoptosis induction, JNK activation, and mitochondrial fission, depend on such ceramide-induced oxidative stress [91–95]. ROS and NO promote mitochondrial fission, which in turn promotes ROS formation through cytochrome c oxidase [96, 97].

In a recent clinical trial, a high-saturated-fat diet increased the serum concentrations of angiotensin-converting enzyme (ACE) independently of weight gain [98]. In the classical renin-angiotensin system (RAS), ACE converts angiotensin I to the active angiotensin II which signals via angiotensin receptors 1 and 2 (AT_1_ and AT_2_) [99] and TLR4 to induce NF-*κ*B activation, mitochondrial fission, and insulin resistance in skeletal muscle cells, vascular smooth muscle cells, and endothelial cells [31, 99–104]. Angiotensin II signaling upregulates xanthine oxidase protein expression and activity in a Nox-dependent manner in endothelial cells [105]. Furthermore, it activates 12-lipoxygenase, whose product, 12-hydroxyeicosatetraenoic acid (12-HETE), induces NF-*κ*B in endothelial cells and aldosterone in adrenal glomerulosa cells [106, 107]. Aldosterone levels increase in humans during obesity, and this hormone correlates with insulin resistance independently of the body mass index [108, 109]. Aldosterone increases superoxide production in endothelial cells though mineralocorticoid receptor- (MR-) mediated activation of Nox and Rac 1 [110]. It also promotes MR-induced de novo ceramide synthesis in these cells [111]. This adrenal hormone readily enters the brain, such that its levels in the brain are directly proportional to its plasma levels in rats [112]. It activates the hypothalamic renin-angiotensin system and associated oxidative stress in hypothalamic neurons [112, 113].

**Figure 3:**
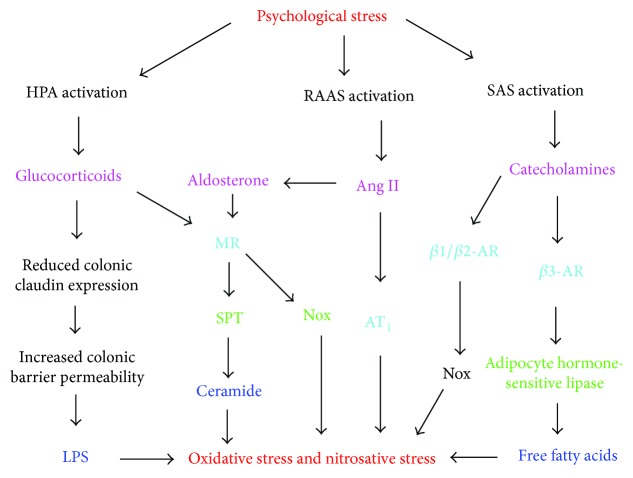
Psychological stress-dependent pathways to oxidative and nitrosative stresses. Psychological stress activates the renin-angiotensin-aldosterone system (RAAS), the hypothalamic-pituitary-adrenal axis (HPA), and the sympathetic adrenomedullary system (SAS), leading to increased availability of angiotensin II (Ang II), aldosterone, glucocorticoids, catecholamines, and free fatty acids which induce oxidative and nitrosative stresses in insulin target cells [109, 117–120, 127, 128]. Glucocorticoids and aldosterone promote de novo ceramide synthesis in endothelial cells and may thereby contribute to plasma ceramides [111, 121–123]. Glucocorticoids also increase colon epithelial barrier permeability and thus increase circulating LPS [124–126]. Angiotensin II, glucocorticoids, aldosterone, and catecholamines upregulate Nox activity in various insulin target cells [105, 110, 130, 132].

Psychological stress (PS) is another major inducer of oxidative stress and insulin resistance [114–116]. This is partly through increased production of aldosterone [109], angiotensin II [117], and glucocorticoids such as corticosterone and cortisol (Figure 3) [118–120]. Glucocorticoids upregulate the expression of SPT and ceramide synthases and thus contribute to ceramide-mediated oxidative stress and insulin resistance [121, 122]. They have a higher affinity for the mineralocorticoid receptor than for the glucocorticoid receptor, and their binding to the former increases Nox expression in adipocytes [123]. PS also contributes to LPS-induced oxidative stress and insulin resistance by promoting colonic barrier permeability and the translocation of bacteria and LPS from the intestinal lumen to the blood [124]. Chronic peripheral administration of corticotropin-releasing factor was demonstrated to cause such colonic barrier dysfunction in rats [125]. This involves glucocorticoid-mediated downregulation of the intestinal epithelial tight junction protein, claudin 1 [126].

Chronic activation of the sympathetic nervous system and the associated increase in catecholamines such as epinephrine and norepinephrine are another hallmark of PS [127]. These catecholamines contribute to insulin resistance in the heart by activating *β*-adrenergic receptors (*β*-AR) [128]. Activation of *β*-AR induces oxidative stress in cardiomyocytes, adipocytes, and endothelial cells, at least partly by *β*2-AR-mediated upregulation of Nox [128–132]. The *β*3-AR activates hormone-sensitive lipase in adipocytes and thus promotes accumulation of free fatty acids and the associated increase in ceramide synthesis and MAPK activation [133]. AR stimulation inhibits adiponectin gene expression in adipocytes via protein kinase A [134], and this should further promote ceramide accumulation and ceramide-dependent oxidative stress because adiponectin increases ceramidase activity [135].

**Figure 4:**
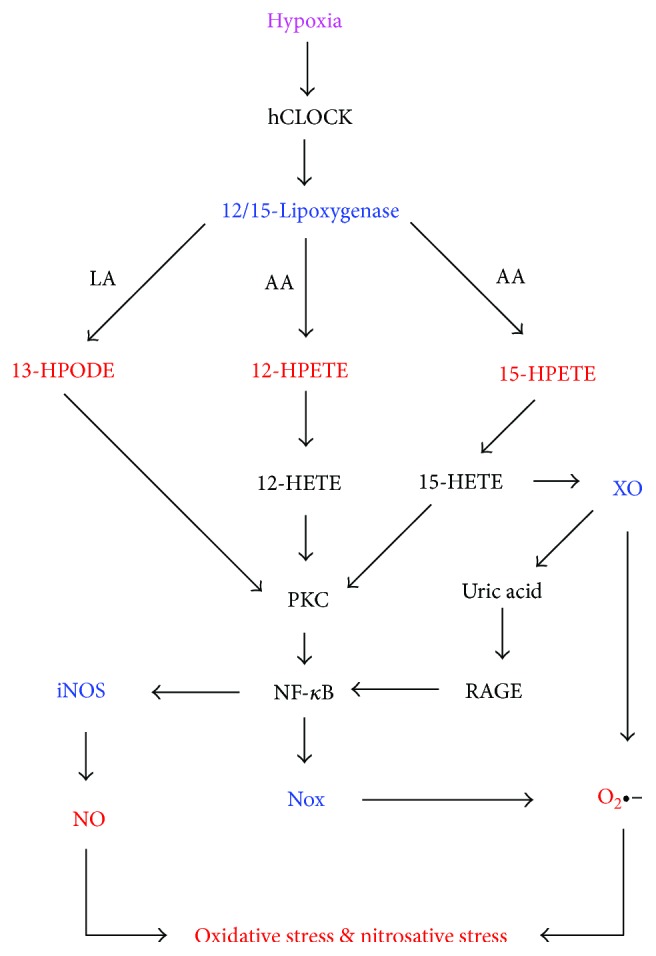
Pathways for the hypoxia-induced generation of oxidative and nitrosative stresses through the human circadian locomotor output cycle protein kaput- (hCLOCK-) mediated 12/15-lipoxygenase activation [143–150]. 12/15-Lipoxygenase catalyses the production of ROS in the form of 13-hydroperoxy-octadecadienoic acid (13-HPODE) from linoleic acid (LA) or 12-hydroperoxy-eicosatetraenoic acid (12-HPETE) and 15-hydroperoxy-eicosatetraenoic acid (15-HPETE) from arachidonic acid (AA). 13-HPODE, 12-HPETE, and 15-HPETE are reduced to the corresponding hydroxy derivatives 13-HODE, 12-HETE, and 15-HETE, respectively. 13-HPODE, 12-HETE, and 15-HETE activate PKC isoforms [148, 151, 152], which promote activation of NF-*κ*B, NADPH oxidase (Nox) isoforms, and inducible nitric oxide synthase (iNOS) [30, 144–150]. iNOS and Nox produce nitric oxide (NO) and superoxide anions (O_2_^·−^), respectively, which undergo enzymatic and nonenzymatic reactions that lead to the formation of other ROS such as hydrogen peroxide and singlet oxygen, as well as the RNS, peroxynitrite [30]. 15-HETE activates xanthine oxidase (XO) [154], which catalyses the formation of both superoxide anions and uric acid, and the latter signals via the receptor for advanced glycation end products (RAGE) to activate NF-*κ*B [34].

Mountain climbing or obstructive sleep apnea (OSA) induces insulin resistance, and this is associated with hypoxia-induced oxidative and nitrosative stresses [136, 137]. OSA worsens during periods of rapid weight gain [138]. Chronic asthma also induces intermittent hypoxia [139], and an association between asthma and insulin resistance was demonstrated in children and adolescents [18, 140, 141]. Many mechanisms have been reported to be involved in hypoxia-induced oxidative and nitrosative stresses. During the switch from normoxia to hypoxia, a burst of superoxide formation occurs at mitochondrial complex 1 due to deactivation of this complex in cells such as endothelial cells and neurons [142]. Hypoxia also increases superoxide formation at mitochondrial complex III [143]. In human umbilical endothelial cells, hypoxia was found to induce expression of the human circadian locomotor output cycle protein kaput (hCLOCK), which promoted the production of ROS, which in turn promoted Rhoa and NF-*κ*B signaling [144]. Hypoxia upregulates 12/15-lipoxygenase, whose metabolites, namely, 13-hydroperoxyoctadecadienoc acid (13-HPODE), 12-hydroxyeicosatetraenoic acid (12-HETE), and 15-hydroxy-eicosatetraenoic acid (15-HETE), activate NF-*κ*B, iNOS, and mitochondrial oxidative stress in endothelial cells, cardiomyocytes, smooth muscle cells, neurons, and adipocytes [145–151]. Since 13-HPODE, 12-HETE, and 15-HETE activate PKC isoforms [149, 152, 153], the 12/15-lipoxygenase-dependent, hypoxia-induced oxidative and nitrosative stresses may follow the pathway outlined in Figure 4.

As indicated in this figure, 15-S-HETE also activates xanthine oxidase (XO) in endothelial cells [154]. Such increase in XO activity occurs during hypoxia [155] and leads to increased uric acid (UA) formation in lowlanders at high altitude [156]. Uric acid is a promoter of oxidative stress via the RAGE receptor in endothelial cells [34]. During intermittent hypoxia, XO-derived ROS activate Nox2 [157]. In addition, hypoxia increases catecholamine production [158] and, like psychological stress, has been reported to induce mucosal barrier failure and endotoxemia in rats and primates [159, 160]. However, a recent study in humans found that hypoxia increased gut inflammation but not gut permeability [161].

Long-term exposure to traffic-related air pollution was found to be positively associated with insulin resistance in children [162]. Particulate matter, ozone, nitrogen oxides, and transition metals are among the potent oxidants in polluted air that induce endogenous ROS formation and oxidative stress [163]. Cadmium, a heavy metal pollutant from industrial plants, which makes its way into the food chain and induces oxidative stress was recently found to be positively associated with insulin resistance [164].

### 2.2. Oxidative Stress Produces Carbonyl Stress and Vice Versa

Decomposition of lipid hydroperoxides produces reactive carbonyl compounds including acrolein, glyoxal, methylglyoxal, malondialdehyde, 4-hydroperoxy-2-nonenal, 4-hydroxy-2-nonenal (HNE), 4-oxo-2-nonenal, 2,4-decadienal, and 9-oxo-nonanic acid [165]. Elevated formation of such products constitutes carbonyl stress. Thus, oxidative stress, through increased production of lipid hydroperoxides, promotes carbonyl stress. As mentioned in the previous section, methylglyoxal- and glyoxal-derived AGEs promote oxidative stress via the RAGE receptor. Likewise, 4-HNE, one of the predominant lipid-derived aldehydes formed in insulin-responsive cells during high-fat or high-glucose diets, promotes the formation of reactive oxygen and nitrogen species [166]. Cholesterol secosterol aldehydes, which are produced via the reaction of cholesterol with singlet oxygen or ozone [30, 167], increase oxidative stress by inactivating catalase and thus promoting the accumulation of hydrogen peroxide and lipid hydroperoxides [168].

### 2.3. Oxidative and Carbonyl Stresses Promote Endoplasmic Reticulum Stress and Vice Versa

Noncytoplasmic and nonmembrane proteins synthesized at the rough endoplasmic reticulum (ER) undergo translocation into the ER lumen, where calcium-dependent molecular chaperones assist their folding into the correct tertiary structures [169]. The ER calcium transporter, sarco- (endo-) plasmic reticulum Ca^2+^ ATPase (SERCA), pumps calcium ions into this organelle and thereby promotes the activity of the molecular chaperones [170, 171]. The reversible S-glutathionylation of SERCA thiols by NO and peroxynitrite increases SERCA activity, but the irreversible sulfonation of these thiols by ROS such as hydrogen peroxide and singlet oxygen causes its inactivation [30, 170–172]. The ensuing accumulation of unfolded or misfolded proteins in the ER constitutes ER stress [173]. Carbonylation by aldehydes such as acrolein, methylglyoxal, glyoxal, and HNE also reduces SERCA activity [174, 175]. ER stress leads to enhanced Nox 4 activity in the ER, resulting in increased hydrogen peroxide formation and oxidative stress [176]. Such increased ER oxidative stress promotes calcium efflux from the ER and calcium influx into the mitochondria, which induces mitochondrial ROS production and oxidative stress [30, 176]. Thus, there is a vicious cycle between ER stress and oxidative stress [30, 177].

### 2.4. Oxidative, Carbonyl, and Nitrosative Stresses Generate Genotoxic Stress

Oxidative stress, carbonyl stress, and nitrosative stress contribute to genotoxic stress by availing genotoxic reactive oxygen, carbonyl, and nitrogen species that modify DNA. ROS react with the nitrogenous bases in DNA to induce a variety of base modifications. One of the most common of such modifications is the conversion of guanine to 8-oxo-7,8-dihydroguanine (8-oxoG), whose levels in urine have been suggested to be a marker of whole-body oxidative stress [178, 179]. 8-oxoG is most readily formed by singlet oxygen, although the hydroxyl radical also contributes to its formation [180, 181]. Mitochondrial DNA is exposed to singlet oxygen generated through mechanisms such as the reaction of peroxynitrite with hydrogen peroxide or cytochrome c-mediated conversion of cardiolipin hydroperoxide to triplet carbonyls in the mitochondria [30]. DNA-damaging hydroxyl radicals may be generated by the Fenton reaction between DNA-bound Fe2+ and hydrogen peroxide [182]. 8-oxoG undergoes further oxidative modifications, as well as crosslinking with lysine to generate protein-DNA adducts [183]. The reaction of singlet oxygen or hydroxyl radicals with deoxyribose in DNA generates single-strand breaks, but double-strand breaks can be generated when the single-strand breaks occur in close proximity [178, 184]. Peroxynitrite induces single-strand breaks in DNA through deoxyribose oxidation or via the formation of 8-nitroguanine [185]. Reactive carbonyl compounds derived from the decomposition of lipid hydroperoxides react with DNA bases to form exocyclic propano- and etheno-DNA adducts, as recently reviewed [186]. The glycoxidation of histone proteins by glyoxal and methylglyoxal promotes the oxidative generation of DNA strand breaks [187]. The formation of hydrogen peroxide and singlet oxygen during protein glycoxidation [74] may explain this phenomenon. Genotoxic stress in turn promotes oxidative stress (Section 3.4).

## 3. Mechanisms of the Inhibition of Insulin Signaling by Cellular Stresses

### 3.1. Oxidative Stress

**Figure 5:**
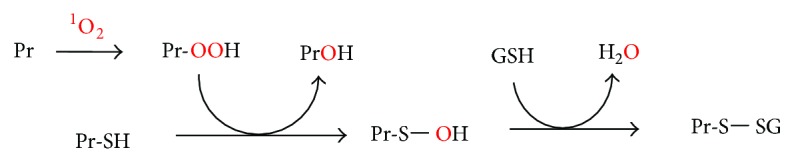
Mechanism of singlet oxygen-mediated protein glutathionylation. Reaction of ^1^O_2_ with a protein (Pr) on residues such as tryptophan and histidine generates a protein hydroperoxide (Pr-OOH). Reaction of Pr-OOH with cysteine residues in other proteins (Pr-SH) converts the latter to sulfenic acids (Pr-SOH) [189], which readily react with glutathione (GSH) to form glutathionylated proteins (Pr-S-SG) [190, 191].

The oxidative modifications of biomolecules including lipids, nucleic acids, and proteins contribute to insulin resistance. Some common types of oxidative protein modifications include hydroperoxidation, glutathionylation, and sulfonation. As shown in Figure 5, proteins (Pr) react with singlet oxygen to form protein hydroperoxides (Pr-OOH). The latter is relatively long lived and inactivates enzymes even when singlet oxygen is no longer in the system [188, 189]. Pr-OOHs react with thiol (-SH) groups in other proteins to form hydroxy proteins (Pr-OH) and protein sulfenic acids (Pr-S-OH), and the latter readily reacts with glutathione (GSH) to form glutathionylated proteins (Pr-S-SG) [190–192]. Hydrogen peroxide also induces protein glutathionylation, analogously to protein hydroperoxides, but the latter is more reactive [189]. Sulfenic acids (Pr-SOH) react further with H_2_O_2_ to form sulfinic acids (Pr-SO_2_H), which react with H_2_O_2_ to form sulfonic acids (Pr-SO_3_H) [190]. The reaction of superoxide radicals with thiols may also lead to the conversion of the latter to sulfonates via persulphenyl derivatives [193]. Ozone or ozone-like oxidants have been suggested to be formed in biological systems [74, 167]. Ozone largely converts thiolate ions to sulfonates [194] and was postulated to be an important contributor to the conversion of methionine sulfoxide to methionine sulfonate [195].

At low levels, ROS including H_2_O_2_ and singlet oxygen stimulate insulin signaling by the PI3K-Akt pathway through inhibition of protein tyrosine phosphatase 1B (PTP1B) which dephosphorylates the insulin receptor [196, 197]. On the other hand, a high concentration of H_2_O_2_ was found to induce insulin resistance in hepatocytes, and systemic removal of hydrogen peroxide improved insulin resistance in obese mice [196, 198]. ROS activate stress-sensitive kinases which reduce insulin signaling [199], and it was proposed that at high H_2_O_2_ concentrations, JNK activation outweighs PTP1B inactivation [196].

Activation of JNK and p38 by ROS occurs through the modification of their regulatory proteins. For example, MAPK phosphatase 1 deactivates JNK and p38 MAPK by dephosphorylation, but glutathionylation targets this phosphatase for proteosomal degradation [199].

Protein-protein interaction between glutathione S-transferase P (GSTP) and JNK keeps the latter in an inactive state, but oxidative modification of the former breaks this interaction and activates JNK [200, 201]. Another protein whose oxidative modification promotes insulin resistance is thioredoxin which, in the native state, binds to and inactivates apoptotic signaling kinase 1 (ASK1), an upstream activator of both JNK and p38 pathways [202, 203]. While the release of thioredoxin from ASK would also allow the former to act as a thiol-reducing antioxidant, oxidative stress promotes the p38 MAPK- and FOXO-dependent expression of thioredoxin-interacting protein (TXNIP) [204] and transfer of the latter from the nucleus to the cytoplasm and mitochondria, where it binds to thioredoxin 1 and thioredoxin 2, respectively, and this aggravates oxidative stress [204, 205]. Increased oxidative stress also favors the activatory binding of TXNIP to the NLRP3 inflammasome [205], a key contributor to insulin resistance as described in Section 4.5.

Several studies have reported that the inhibition of glycolysis in muscle cells induces insulin resistance [206–208], for example, through a compensatory increase in lipid uptake [208]. Protein peroxides generated by singlet oxygen inhibit the glycolytic enzyme glyceraldehyde-3-phosphate dehydrogenase (GPD) [209]. Besides reduced glycolysis, GPD inhibition enhances the conversion of dihydroxyacetone phosphate to methylglyoxal [210], which is one of the major reactive carbonyls contributing to insulin resistance (Section 3.3).

Reactive oxygen species such as H_2_O_2_ promote Ser637 dephosphorylation of the GTPase, dynamin-related protein 1 (Drp1), resulting in translocation of the latter to the mitochondria, where its polymerization into a ring-like structure induces mitochondrial fission [211], an important contributor to insulin resistance and oxidative stress [94, 95].

Perhaps one of the greatest contributions of oxidative stress to insulin resistance is that it generates metabolites that create positive feedback loops for potentiation of TLR4, RAGE, and other signaling pathways associated with activation of NF-*κ*B and insulin signal-inhibiting serine kinases such as PKC, IKK, JNK, and p38 MAPK. For example, oxidative stress leads to the oxidation of low-density lipoproteins (LDL), and oxidized LDL (oxLDL) signals via Lox-1, RAGE, and Fas receptors to activate NF-*κ*B and MAPKs as recently reviewed [30], and the plasma concentration of oxLDL is an independent risk factor for insulin resistance [212].

p38 MAPK promotes the expression of aldose reductase (ALDR) [213], which is subsequently activated by oxidative modification [214], and makes a major contribution to insulin resistance [215]. ALDR reduces HNE-glutathione adduct (GS-HNE) to glutathionyl-1,4-dihydroxynonene (GS-HN), which activates phospholipase C, leading to DAG formation and the activation of PKC, MAPKs, and NF-*κ*B (Figure 2) [166, 216]. DAG oxidation also contributes to PKC-dependent signaling, since DAG hydroperoxide is a more potent activator of PKC than unoxidized DAG [217]. In the presence of excess glucose, ALDR catalyses the first reaction of the polyol pathway, which leads to the production of both DAG and AGES, thus contributing to signaling via both TLR4 and RAGE [218]. The role of ALDR in potentiating LPS-TLR4 signaling via PKC is evidenced by findings that its inhibition alleviates endotoxin-induced inflammatory diseases [216, 219, 220].

Mammalian xanthine dehydrogenase (XDH) is reversibly converted to xanthine oxidase (XO) by oxidative modification of specific cysteine residues [221]. As already mentioned, lipoxygenase-mediated 15-HETE formation during hypoxia activates XO. Fructose metabolism in hepatocytes is associated with increased XO-mediated conversion of AMP to uric acid [85, 222–224], which promotes insulin resistance through RAGE, TLR4, NF-*κ*B, Nox, mitochondrial oxidative stress, ER stress, and skeletal muscle atrophy [30, 34, 223–227].

Oxidative stress promotes ceramide synthesis even independently of SPT and ceramide synthases. Singlet oxygen converts sphingomyelin to ceramide, even in protein-free liposomes [228]. In glioma cells, superoxide promotes ceramide formation through activation of neutral sphingomyelinase [229]. Sphingomyelinase inhibition reduces intramyocellular ceramide and protects muscle cells from insulin resistance [230, 231].

H_2_O_2_ downregulates the expression of carnitine palmitoyl transferase 1 (CPT1), acyl COA oxidase (ACOX), and peroxisome proliferator-activated receptor-alpha (PPAR-*α*) in hepatocytes [232] and PPAR-*γ* in endothelial cells [233]. CPT1 and ACOX are involved in fatty acid oxidation and reduction of DAG and ceramide levels [232, 234, 235]. PPAR-*α* reduces oxidative stress by upregulating superoxide dismutase and catalase expression and inhibiting NF-*κ*B activity [232, 236, 237]. PPAR-*γ* inhibits NF-*κ*B and upregulates the expression of adiponectin, an adipokine that improves insulin sensitivity [238–240].

### 3.2. Nitrosative Stress

The importance of nitrosative stress in insulin resistance is evidenced by reports that inhibition of iNOS or peroxynitrite in various cell types prevents insulin resistance [41–43]. Peroxynitrite decomposes into radicals that cause inhibitory tyrosine nitration of proteins in the insulin signaling pathway [70]. The reaction of peroxynitrite with proteins generates thyl radicals and sulfenates, leading to protein glutathionylation [192]. Nitrosoglutathione causes glutathionylation and inhibition of GADPH [241]. Peroxynitrite contributes to palmitate-induced DNA damage and inflammasome activation [23, 242]. It also induces ceramide formation in endothelial cells [243]. Nevertheless, the effects of peroxynitrite are mediated to some extent by ROS since peroxynitrite-derived radicals can initiate free radical lipid peroxidation [244], and the reaction of peroxynitrite with hydrogen peroxide generates singlet oxygen [30, 71]. Thus, iNOS and NO donor-induced IRS-1 degradation in skeletal muscle cells was accentuated by concomitant oxidative stress [245].

### 3.3. Carbonyl/Electrophilic Stress

Reactive carbonyl species contribute to insulin resistance in various ways. Methylglyoxal, HNE, and cholesterol secosterol aldehydes participate in the generation of oxidative stress and associated NF-*κ*B and MAPK activation (Sections 2.1, 2.2, and 3.1). In addition, methylglyoxal adducts insulin and inhibits the latter's proper interaction with the insulin receptor [246]. Protein-HNE adducts correlate with intramyocellular lipid content and the severity of insulin resistance in humans [247]. HNE forms Michael adducts with His196 and Cys311 of Akt2 and thus inhibits downstream phosphorylation of Akt substrates such as glycogen synthase kinase 3*β* (GSK3*β*) and MDM2, resulting in the activation of the former and inhibition of the latter [247]. GSK3*β* inhibits glycogen synthase and IRS and thus prevents both glycogen synthesis and glucose transport [248–250]. It also promotes hepatic gluconeogenesis by an unknown mechanism [251] and contributes to the dysregulation of the Nrf2 antioxidant response [252]. MDM2 is a negative regulator of the p53 protein, which promotes insulin resistance as discussed in Section 3.4.

Human adipocytes and white adipose tissue express the full enzymatic machinery required for the synthesis and metabolism of asymmetric NGNGdimethylarginine (ADMA) which uncouples NOS and thus promotes ROS formation, increases TLR4 expression, decreases IRS-1 and GLUT-4 expression, and inhibits IRS-1 tyrosine phosphorylation and GLUT-4 translocation [253–256]. Plasma levels of ADMA increase during oxidative stress, mainly due to decreased expression and activity of the ADMA-degrading enzyme, dimethylarginine dimethylaminohydrolase (DDAH) [255–259]. HNE downregulates DDAH-1 expression through an miR-21-dependent mechanism [259].

### 3.4. Genotoxic Stress

Increased oxidative DNA damage determined as serum 8-hydroxy-2-deoxy-guanosine (8-OHdG) was found in lean normoglycemic offspring of type 2 diabetics, who are more predisposed to insulin resistance [260]. Similarly, serum level of 8-OHdG was found to be increased in prediabetes [261]. Mice deficient in 8-oxoguanine DNA glycosylase (the enzyme that performs base excision repair of DNA by cleaving 8-oxoG and other modified bases) were found to be prone to insulin resistance upon high-fat feeding [262]. Mitochondrial DNA damage promotes palmitate-induced insulin resistance mainly by increasing mitochondrial oxidative stress, ER stress, JNK activation, and apoptosis, since overexpression of DNA glycosylase/apurinic/apyrimidinic lyase (hOGG1) in the mitochondria of skeletal muscle cells abrogated these effects [263, 264]. Interestingly, prevention of mitochondrial DNA damage in cardiomyocytes exposed to angiotensin II prevented mitochondrial superoxide production in these cells [265]. In the latter study, mtDNA damage was found to cause impairments in mitochondrial protein expression, cellular respiration, and complex 1 activity prior to enhanced mitochondrial superoxide production. In addition, oxidized mitochondrial DNA released into the cytoplasm during apoptotic signaling activates the NLRP3 inflammasome [266].

### 3.5. Endoplasmic Reticulum Stress

Endoplasmic reticulum stress contributes to insulin resistance by promoting oxidative stress, especially mitochondrial oxidative stress and the resultant carbonyl and genotoxic stresses (Section 2.3) and ceramide synthesis [46], as well as by triggering the unfolded protein response [267], inflammasome activation [268], and apoptosis (Section 4.4).

## 4. Inhibition of Insulin Signaling by Cellular Stress Responses

### 4.1. The Ubiquitin-Proteosome Pathway

The ubiquitin-proteosome pathway (UPP) is the major cytosolic mechanism for the selective degradation of damaged proteins, such as oxidatively modified proteins, whereby the damaged proteins are conjugated to multiple ubiquitin molecules and then degraded by the 26S proteasome [269]. This system is upregulated by mild and moderate oxidative stress and is required for cells to cope with oxidative stress [269]. On the other hand, UPP-mediated degradation of the NF-*κ*B inhibitor, iKB, causes activation of NF-*κ*B [270]. NF-*κ*B promotes oxidative stress and induces expression of proinflammatory cytokines including TNF-*α* and IL-6 which, via the JAK-STAT pathway, upregulate expression of suppressors of cytokine signaling (SOCS) proteins such as SOCS1 and SOCS3 [271–273]. Association of the SOCS proteins with IRS targets the latter for degradation by the ubiquitin-proteosome pathway in multiple cell types [271–273]. Accordingly, palmitate-induced insulin resistance in L6 myotubes was found to be dependent on constitutive phosphorylation of STAT 3 and the associated increase in protein expression of SOCS 3 [274], and the ubiquitination and proteosomal degradation of IRS-1 and Akt was demonstrated to contribute to palmitate or NO donor-induced insulin resistance in HepG2 cells and skeletal muscle cells, respectively [245, 275]. Moreover, increased SOCS1/SOCS3 expression during uveitis induces insulin resistance in neuroretina [276], and SOCS3 overexpression is responsible for the induction of insulin resistance in mice infected with hepatitis C virus [277].

Several factors contribute to increased UPP during the pathogenesis of insulin resistance. For example, the 15-lipoxygenase product, 15-HETE, induces the expression of key enzymes of the UPP pathway [150]. Inhibition of the adipose tissue ERK1/2 pathway during a high-fat diet was reported to enhance the UPP-mediated degradation of adiponectin [278], although contradictory results that ERK activity increases in hypertrophic adipocytes have also been obtained [279]. Increased HNE activity during a high-fat diet enhances the ubiquitin-proteosome-mediated degradation of adiponectin [280]. This is detrimental since adiponectin improves insulin sensitivity by (i) upregulating ceramidase activity to decrease ceramide levels [135]; (ii) increasing the levels of tetrahydrobiopterin (BH4), which lowers hepatocyte gluconeogenesis by activating AMP-activated kinase (AMPK) in an eNOS-dependent process [281]; and (iii) upregulating the silent information regulator 1 (SIRT1), a nicotinamide adenine dinucleotide-dependent histone deacetylase [282].

SIRT1 plays a role in the reduction of oxidative stress through increased expression of superoxide dismutase and catalase subsequently to FOXO4 deacetylation [283]. It also activates AMP-activated kinase (AMPK), which promotes insulin sensitivity by inhibiting PKC isoforms and the associated NF-*κ*B activation, oxidative stress, ER stress, and apoptosis [284–291]. AMPK also induces mitochondrial biogenesis, which limits endothelial cell dysfunction, for example, in response to angiotensin II signaling [292]. Phosphorylation of SIRT1 by JNK1 primes SIRT1 for ubiquitination and degradation, and persistent JNK1 activation in obesity causes severe hepatic SIRT1 degradation [293]. SIRT1 reduction is detrimental to insulin signaling in various tissues including liver, skeletal muscle, and adipose tissues [294–296]. AMPK1 is also diminished in insulin-resistant individuals, and pharmacological agents that activate it, such as metformin, improve insulin signaling [290].

Increased mitochondrial DNA methylation in NADH dehydrogenase 6 (ND6) and displacement loop (D-loop) regions significantly correlates with insulin resistance, and SIRT1 deregulation was suggested to be involved in such epigenetic changes [297]. Since AMPK-mediated phosphorylation results in inhibition of DNA methyl transferase 1 (DNMT1) [298], UPP-mediated SIRT1 downregulation may induce such epigenetic changes through AMPK inhibition. Inflammasome activation might also contribute to this process by promoting DNMT1 expression (see Section 4.5). On the other hand, one study recently found that oxidative stress downregulated DNMT1 isoform 3, the isoform that is responsible for mitochondrial DNA methylation [299]. Further studies are necessary to resolve this apparent contradiction.

The UPP may especially be relevant in skeletal muscle insulin resistance by contributing to skeletal muscle atrophy, which occurs in two steps, namely, (i) the release of myofilaments from the sarcomere by cysteine proteases such as calpain and caspases and (ii) UPP-mediated degradation of the myofilament fragments [300, 301]. Calpain activation may in turn rely on the release of calcium from the ER during ER stress. Calpain activation in the skeletal muscle results in inhibited Akt activity, which in turn results in the activation of Foxo transcription factors that activate expression of components of the ubiquitin-proteosome system involved in muscle protein degradation [301]. Muscle atrophy per se has been associated with insulin resistance due to a decline in muscle oxidative capacity [302]. Exposure of C2C12 myotubes to 25 *μ*M H_2_O_2_ induced calpain-dependent atrophy without cell death [300]. Exposure of C2 myotubes to peroxynitrite induced degradation of the myosin heavy chain muscle through activation of p38 MAPK and upregulation of the muscle-specific E3 ubiquitin ligases atrogin-1 and MuRF1 [303]. Increased expression of the transforming growth factor-*β* (TGF-*β*) and myostatin, via NF-*κ*B, induces proteosomal degradation of cellular proteins [304, 305], and muscle myostatin mRNA correlates with HOMA2-IR in nondiabetic individuals [306].

While mild or moderate oxidative stress upregulates the ubiquitin-proteosome pathway, severe or sustained oxidative stress inactivates this system, especially the 26S proteasome [269, 307]. This also contributes to insulin resistance since proteosomal dysfunction, characterized by increased levels of carbonylated and ubiquitinated proteins, aggravates oxidative stress and ER stress [308].

### 4.2. The Unfolded Protein Response

ER stress triggers a transcriptional and translational response referred to as the unfolded protein response (UPR), aimed at reducing the translation of global proteins, enhancing the degradation of unfolded proteins, and increasing the transcription of genes that enhance the protein folding capacity of the ER [267]. The double-stranded RNA-dependent protein kinase- (PKR-) like ER kinase (PERK), the inositol requiring kinase 1 (IRE 1), and activating transcription factor 6 (ATF 6) are ER transmembrane proteins that are key components of three different UPR signaling pathways [267]. Details of the signaling events that occur after activation of PERK, IRE1, and ATF 6 have been described elsewhere [267, 309].

All three UPR pathways promote NF-*κ*B activity and oxidative stress [310, 311]. Besides, IRE-1 stimulates ASK-1 and thus activates JNK and p38 MAPK [312]. PERK promotes insulin resistance by (i) activating JNK and p38 MAPK [49, 313]; (ii) phosphorylating FOXO on S298, a site which is not phosphorylated by Akt and whose phosphorylation counteracts the effects of Akt [49]; (iii) downregulating expression of the serine protease prostatin (PRSS8), which regulatorily degrades TLR4 [314]; (iv) inducing the pseudokinase tribble 3 (TRB3), which is increased in the liver of obese mice and humans and contributes to hepatic insulin resistance [49, 315]. TRB3 binds to Akt and prevents insulin-mediated Akt phosphorylation [49, 173, 315].

Low-grade hypothalamic inflammation induced by TNF-*α* was found to reduce oxygen consumption and the expression of thermogenic proteins in adipose tissue and skeletal muscles, and this was associated with insulin resistance in a rat model [316]. PERK, to a lesser extent, ATF, and IRE upregulate the C/EBP homologous protein (CHOP) [317], which downregulates cAMP-induced upregulation of uncoupling protein 1 in adipocytes and thus prevents adaptive thermogenesis [318].

### 4.3. The DNA Damage Response

The DNA damage response is a complex mechanism to detect DNA damage, including strand breaks or base modifications, promote their repair, and in case of excessive damage to activate cell death pathways [319]. Sensing of double-strand breaks by the Mre11-Rad50-Nbs1 (MRN) complex leads to the activation of ataxia telangiectasia mutated (ATM) and subsequent activation of DNA damage checkpoints [319]. ATM activates NF-*κ*B following DNA damage [320]. Checkpoints induce changes in telomeric chromatin, recruit DNA repair proteins to sites of DNA damage, and induce cell death by apoptosis [321]. One of the checkpoints, Chk2, promotes transcription of genes involved in DNA repair and phosphorylates tumor suppressor p53, thereby reversing inhibition of the latter by MDM2 [319]. Chk2 also phosphorylates MDM4 and thereby reduces p53 degradation [319]. Such activation of the p53 pathway is upregulated in adipose, endothelial, hepatic, and skeletal muscle tissues of obese rodents and humans [322–326]. Although this protein exhibits antioxidant activity at low levels of oxidative stress, it becomes prooxidant at higher oxidative stress levels, through activation of NF-*κ*B [327] and promotion of ceramide synthesis by upregulating ceramide synthases [328]. p53 promotes cellular senescence in adipose tissue, and this is associated with increased production of proinflammatory cytokines, which promote adipose tissue infiltration by neutrophils and macrophages, and systemic insulin resistance [322–326]. Many other metabolic effects of p53 are opposed to insulin signaling, as has been exhaustively reviewed [322, 327], including but not limited to JNK activation; apoptosis; repressed expression of the insulin receptor and glucose transporters 1, 3, and 4; enhanced transcription of phosphatase and tensin homologue (PTEN) which reduces phosphorylation of P13K and Akt; Akt degradation; glycolysis inhibition; and downregulated expression of peroxisome proliferator-activated receptor-*γ* coactivator-1*α* (PGC-1*α*) in the skeletal muscle, leading to the reduction in mitochondrial biogenesis and energy consumption. Reduced mitochondrial biogenesis leads to a lower capacity for fatty acid metabolism in skeletal muscle cells and accumulation of intramyocellular lipids including DAG which is a major contributor to skeletal muscle insulin resistance [329, 330].

### 4.4. Apoptosis

Apoptosis is a type of programmed cell death in response to cellular damage or other physiological cues, characterized by controlled autodigestion of the cell by caspases [331, 332]. It is regarded as extrinsic when it involves death receptors such as CD95 (Fas) or intrinsic if it occurs independently of such receptors, and both forms of apoptosis were found to be involved in insulin resistance in a mouse model [55]. Caspase-8 and caspase-9, respectively, act as initiator caspases for extrinsic and intrinsic apoptosis, and each initiator caspase starts off a cascade for the activation of executer caspases, which degrade key cellular proteins [333].

Oxidative stress promotes Fas ligand expression in various cell types and thus promotes extrinsic apoptosis [334]. Activation of the Fas receptor by metabolites such as IL-1*β*, oxLDL, singlet oxygen, HNE, or JNK leads to its intracellular recruitment of the adaptor protein FADD to form a death-inducing complex (DISC) which activates initiator caspase-8, while the autocatalytic activation of procaspase-9, the initiator of intrinsic apoptosis, requires the assembly of a multiprotein complex, the apoptosome, which comprises seven copies of heterodimers between apoptotic protease-activating factor 1 (Apaf-1) and cytochrome c [331–335]. Thus, the release of the latter from the mitochondrial membrane into the cytoplasm is a key event in intrinsic apoptosis.

During unresolved ER stress, ER calcium efflux promotes lysosomal membrane permeabilization (LMP) and the release of lysosomal cathepsins, which promote mitochondrial outer membrane permeabilization (MOMP) and the release of cytochrome c from the mitochondrial intermembrane space [331, 332]. Localization of p53 protein on the lysosomal membrane upon sustained DNA damage also contributes to LMP [336]. Activated JNK contributes to apoptosis in various ways including (i) inducing the expression of proapoptotic genes through transactivation of c-jun or p53, (ii) phosphorylating the BH3-only family of Bcl2 proteins which antagonize the antiapoptotic activity of Bcl2 or Bcl Xl, and (iii) activating Bim, a BH3-domain-only protein which activates Bax, which in turn promotes MOMP and cytochrome c release [337].

In humans, the progression of nonalcoholic fatty liver disease is associated with increasing apoptosis and insulin resistance in the muscle, liver, and adipose tissue [338]. The link between hepatocyte apoptosis and insulin resistance was demonstrated in a mouse model [56]. Adipocytes of obese mice were found to display a proapoptotic phenotype, and genetic inactivation of the key proapoptotic protein Bid protected against adipose tissue macrophage infiltration and systemic insulin resistance [55]. Prevention of apoptosis prevents palmitate-induced insulin resistance in hypothalamic neurons [339]. Palmitate induces apoptosis and insulin resistance in skeletal muscle myotubes, and cell-permeable effector caspase inhibitors reverse the insulin resistance, indicating that cellular remodeling associated with apoptotic signaling induces insulin resistance [340]. In these cells, caspases inhibit glycolysis, in particular the glycolysis-limiting enzymes phosphofructokinase and pyruvate kinase [340, 341]. In adipocytes, the proapoptotic caspase-3 and caspase-6, which participate in both intrinsic and extrinsic apoptosis, cleave peroxisome proliferator-activated receptor-*γ* (PPAR-*γ*), which results in the nuclear export and cytoplasmic degradation of this transcription factor [342]. Inactivation of PPAR-*γ* in adipocytes leads to downregulation of some genes that are important for insulin sensitivity not only in adipose tissue but also in other tissues such as those of the skeletal muscle. For example, PPAR-*γ* inactivation results in decreased expression of GLUT 4 and decreased secretion of adiponectin [343, 344]. Extensive apoptosis of adipocytes, hepatocytes, and skeletal muscle cells is also expected to contribute to systemic hyperglycemia and hyperglycemia-induced stresses that lead to insulin resistance.

### 4.5. NRLP3 Inflammasome Activation

Interleukin-1*β* (IL-1*β*) is an inflammatory cytokine which activates both myeloid and nonmyeloid cells to produce other inflammatory cytokines and chemokines [345, 346]. Processing of the inactive pro-IL-1*β* into the active IL-1*β* requires the formation and activation of a cytoplasmic multiprotein complex called the inflammasome [58, 345]. One of the most intensively studied inflammasomes is the NLRP3 inflammasome which is expressed by myeloid cells and some nonmyeloid cells such as adipocytes, hepatocytes, endothelial cells, skeletal muscle cells, and aortic smooth muscle cells [345, 347–350]. Components of the NLRP3 inflammasome include the NLRP3 sensor, ASC adaptor, and caspase-1 [345]. Signaling pathways through NF-*κ*B, p38, and ERK1 are involved in the expression of both NLRP3 and pro-IL-1*β* [345, 351–353]. Assembly of the NLRP3 components into the active inflammasome complex occurs in response to “danger signals” including increased intracellular ceramide; RAGE- or IRE*α*-dependent increased expression of thioredoxin-binding protein (TXNIP); oxidation of thioredoxin and its dissociation from TXNIP, allowing the latter to bind to the inflammasome; release of lysosomal cathepsin B as a result of LMP; IRE1-*α*- and PERK-dependent activation of CHOP; and release of oxidized mitochondrial DNA as a result of MOMP [58, 204, 205, 265, 267, 353–359].

IL-1*β* is a ligand for the IL-1 receptor which, like TLR4, signals via MyD88, IL-1 receptor-activated kinases (IRAK1 to 4), IKK, and NF-*κ*B (Figure 2) [57, 58, 360] and should thus potentiate the whole model for insulin resistance in insulin target cells (Figure 2). It downregulates IRS-1 expression and reduces tyrosine phosphorylation of IRS-1 in adipocytes [58, 361]. Preadipocytes release IL-1*β*, which both controls adipocyte differentiation and promotes adipocyte insulin resistance even in the absence of macrophages [58]. IL-1*β* also induces epigenetic changes that promote insulin resistance. For example, it stimulates the expression of DNA methyl transferase 1, which hypermethylates the adiponectin promoter and thereby suppresses the expression of this proinsulin signaling adipokine [362].

While inflammasome assembly, caspase-1 activation, and IL-1*β* processing occur and promote insulin resistance in hepatocytes and mature adipocytes, secretion of IL-1*β* by these cells is controversial [58, 268, 363]. Nevertheless, caspase-1 induces the highly inflammatory pyroptotic death of these cells, and this could contribute to the recruitment and activation of inflammatory myeloid cells such as macrophages that secrete IL-1*β* [268, 363]. In the skeletal muscle, activation of the inflammasome contributes to muscle atrophy through activation of atrogenic genes such as MuRF1 and atrogin 1 [350].

NLRP inflammasome activation in endothelial cells leads to increased IL-1*β* in serum and C-reactive protein (CRP) production by endothelial cells [349]. IL-1*β* stimulates endothelial cell production of chemokines such as monocyte chemoattractant protein-1 (MCP-1) and vascular cell adhesion molecule-1 (VCAM-1) which promote leukocyte-endothelium interactions [349, 364], and this may contribute to the transient migration of neutrophils into adipose tissue that occurs at an early stage of high-fat feeding [365]. This process may be further facilitated by the chemotactic effects of H_2_O_2_ and IL-8 produced by adipocytes [30, 366]. Once in adipose tissue, neutrophils may produce large quantities of chemokines and cytokines including IL-1*β* and IL8, resulting in the recruitment of other immune system cells such as macrophages which sustain chronic inflammation [367, 368].

## 5. Inhibition of Insulin Signaling through the Dysregulation of Cellular Stress Responses

### 5.1. Dysregulation of the Heat Shock Response

The heat shock response, which relies on heat shock proteins such as HSP70, is important for physiological resolution of inflammation [369]. Cellular HSP70, HSP72, and HSP25 protect against insulin resistance in humans by mechanisms involving prevention of JNK phosphorylation and apoptosis [51–53]. Obese, insulin-resistant individuals have reduced expression of HSP72 [51]. In adipocytes, downregulation of HSP expression follows sustained NLRP3 inflammasome activation and the associated caspase-1-mediated cleavage of HuR, an mRNA-binding protein that enhances the expression of SIRT1 [369]. This results in reduced SIRT1-dependent upregulation of the transcription and activity of heat shock factor 1 (HSF1), the transcription factor of heat shock proteins [369].

### 5.2. Autophagy Dysregulation

(Macro)autophagy is a homoeostatic process for the bulk degradation of cytoplasmic components including damaged organelles, misfolded proteins, and oxidized lipids, whereby such components are enclosed into double-membraned vesicles called autophagosomes that subsequently fuse with lysosomes [54, 370–372]. Autophagy-related proteins (Atg) are involved in autophagosome formation, and these are functionally categorized into several units, namely, the Atg1/ULK complex (mammals express Ulk 1/2), the class III phosphatidyl inositol 3-kinase (PI3K) complex, the Atg2-Atg18/WIPI complex, the Atg12 conjugation system, the Atg8/LC3 conjugation system, and Atg9 vesicles [373].

Low levels of ROS induce autophagy [373–375], but higher ROS levels inhibit this response [376]. Obesity, which is associated with oxidative stress, is characterized by inhibited autophagy [377], even in adipose tissue that has elevated expression of autophagy genes [378]. Autophagy inhibition occurs partly due to (i) degradation of autophagy proteins by cell death proteases including calpain 1 and caspases such as caspase-3, caspase-6, and caspase-8 [379], (ii) LMP and the release of cathepsins [379, 380], (iii) SIRT downregulation [381, 382], (vi) inhibition of PPAR-*α* [383], and (vii) increased expression of GSK3*β* [384].

Severe hepatic downregulation of the autophagy gene Atg7 was found to occur in genetic and dietary models of obesity, and this caused insulin resistance through enhanced ER stress [54]. Paradoxically, muscle- or liver-specific knockout of the Atg7 gene protected mice from obesity and insulin resistance by upregulating the expression of fibroblast growth factor (FGF21) [385]. FGF21 improves insulin sensitivity by inhibiting mTORC 1 [385, 386], activating NRf2 antioxidant signaling, suppressing the NF-*κ*B pathway, enhancing adiponectin production, and promoting thermogenesis [387–391]. The apparently contradictory effects of obesity-associated downregulation of Atg7 and genetic knockout of Atg7 on hepatocyte insulin resistance [54, 385] may be better understood by considering that Atg7 knockout prevents obesity [385]. In obesity, but not in the lean state, there is resistance to FGF21, because of downregulation of its receptor machinery, including *β*-klotho protein levels [392–394]. Although klotho is critical for FGF21 function [395], it was recently reported that factors beyond *β*-klotho downregulation are important contributors to adipose tissue FGF21 resistance [396].

### 5.3. Dysregulation of the Nrf2 Antioxidant Response

The nuclear factor erythroid-2-related factor-2 (Nrf2) is the master regulator of antioxidant responses, attenuating both oxidative and electrophilic stresses [397, 398]. Under basal conditions, Nrf2 localizes on the cytoskeleton, where its activity is limited through interaction with Kelch-like ECH-associated protein 1 (Keap1), which targets it for ubiquitination and proteosomal degradation [399]. Modification of cysteine residues in Keap1 by ROS, RNS, or RCCs frees Nrf2 to translocate to the nucleus and induce transcription of antioxidant genes having the antioxidant response element in the promoter region [397, 400]. Nevertheless, Nrf2 levels were found to be lower in prediabetic and diabetic patients than in healthy subjects [401], and short-term treatment of high-fat diet-fed mice with curcumin was found to improve insulin sensitivity through attenuating Nrf2 signaling defect [402].

Suppression of Nrf2 activity may be partly due to the direct interaction of p53 with ARE-containing promoters [403]. ERK activation was reported to induce Nrf2 suppression and insulin resistance in cardiomyocytes exposed to hydrogen peroxide [404], but an opposite effect of ERK activation was reported in HepG2 cells exposed to methylglyoxal [405]. In neuronal cells exposed to H_2_O_2_, GSK3*β* activation was shown to be responsible for the cytoplasmic accumulation of Nrf2 [251]. This may involve H_2_O_2_-mediated activatory phosphorylation of tyrosine 216 of GSK3*β*, and the latter phosphorylates the tyrosine kinase Fyn, which then translocates to the nucleus, and phosphorylates tyrosine 568 of Nrf2, leading to Nrf2 export from the nucleus [406].

Nrf2 antioxidant response is also downregulated by cortisol [407], whose production is increased during psychological stress [117]. Obesity is associated with higher adipose tissue expression of 11*β*-hydroxysteroid dehydrogenase type 1 (11*β*HSD1), an enzyme that converts cortisone to active cortisol [408, 409]. Cortisol is a ligand for the mineralocorticoid receptor, whose expression increases in obesity [122].

Notably, Nrf2 is Janus-faced, and its overexpression was found to worsen insulin resistance in mice [410]. Nrf2-knockout mice on a long-term high-fat diet had increased FGF21 expression and better insulin sensitivity than wild-type mice on the same diet, and overexpression of Nrf2 in ST-2 cells was found to decrease insulin sensitivity associated with decreased FGF21 mRNA levels and activity [411]. Nrf2 overexpression may occur during autophagy blockade, which is associated with an increase in the cellular levels of p62 [412, 413]. p62 normally participates in autophagosome formation and undergoes lysosomal degradation with the contents of the autophagosome [412, 413]. However, during autophagy blockade, it sequesters Keap1 into autophagosomes, leading to stabilization and overactivation of Nrf2 by the so called noncanonical pathway [412]. Thus, for beneficial effects, the level of Nrf2 activation needs tight regulation.

## 6. Conclusion

There is substantial literature in support of the hypothesis that insulin resistance develops from a coordinated interplay between various cellular stresses and stress responses that develop upon the exposure of insulin-responsive cells to hypoxia, excess sugars or certain types of fatty acids, environmental pollutants, or hormones released during psychological stress and obesity. This knowledge will help in the design of better strategies for the prevention and management of insulin resistance.
